# Parental and child factors associated with inhalant and food allergy in a population-based prospective cohort study: the Generation R Study

**DOI:** 10.1007/s00431-019-03441-5

**Published:** 2019-08-15

**Authors:** Nicolette W. de Jong, Niels J. Elbert, Sara M. Mensink-Bout, Johanna P. M. van der Valk, Suzanne G. M. A. Pasmans, Vincent W. V. Jaddoe, Johan C. de Jongste, Roy Gerth van Wijk, Liesbeth Duijts

**Affiliations:** 1 Department of Internal Medicine, Division of Allergology, Erasmus Medical Center, Room Gk 323, P.O. Box 2040, 3000 CA Rotterdam, The Netherlands; 2 The Generation R Study Group, Erasmus Medical Center, Rotterdam, The Netherlands; 3 Department of Dermatology, Erasmus Medical Center, Rotterdam, The Netherlands; 4 Department of Pediatrics, Erasmus Medical Center, Rotterdam, The Netherlands; 5 Department of Pediatrics, Division of Respiratory Medicine, Erasmus Medical Center, Rotterdam, The Netherlands; 6 Department of Epidemiology, Erasmus Medical Center, Rotterdam, The Netherlands; 7 Department of Pediatrics, Division of Neonatology, Erasmus Medical Center, Rotterdam, The Netherlands

**Keywords:** Atopic diseases, Birth cohort, Children, Risk factors

## Abstract

The prevalence of allergic diseases in children is markedly increasing to epidemic proportions. The aim of this study is to describe the presence and examine associated parental and child characteristics of allergic sensitization and physician-diagnosed allergy in Dutch children at age 10 years. This study among 5471 children was performed in a population-based prospective cohort from fetal life onwards. Allergic sensitization was measured by skin prick tests. Physician-diagnosed allergy and parental and child characteristics were collected by questionnaires. In children aged 10 years, inhalant and food allergic sensitization was present in 32.2% and 7.1%, and physician-diagnosed inhalant and food allergy in 12.4% and 2.3%. Maternal and paternal history of allergy, eczema or asthma was associated with increased risks of physician-diagnosed inhalant allergy (aOR (95% CI) 1.44 (1.23–1.70) and 1.59 (1.30–1.94), respectively), but not with food allergy. Asthma and eczema ever at age 10 years were associated with increased risks of physician-diagnosed inhalant allergy (4.60 (3.55–5.96) and 2.42 (1.94–3.03), respectively). Eczema ever at age 10 years was associated with an increased risk of physician-diagnosed food allergy (5.78, 3.04–9.52), with the highest risk of cashew (7.36, 3.20–16.94) and peanut (5.58, 3.08–10.10) food allergy.

*Conclusions*: We found strong effects of parental history of allergy, eczema or asthma on the presence of physician-diagnosed inhalant allergy in children at age 10 years. Eczema ever at age 10 years was a strong risk factor for the development of physician-diagnosed inhalant and food allergy.

**Table:** 

**What is Known:** *• The prevalence of allergic diseases in children has markedly increased.* *• Early-life influences are critically important in the development of allergic diseases.*	
**What is New:** *• Maternal and paternal history of allergy, eczema or asthma is associated with increased risks of physician-diagnosed inhalant allergy but not with food allergy.* *• Eczema ever at age 10 years is associated with an increased risk of physician-diagnosed food allergy, with the highest risk for cashew and peanut food allergy.*

**Electronic supplementary material:**

The online version of this article (10.1007/s00431-019-03441-5) contains supplementary material, which is available to authorized users.

## Introduction

The prevalence of allergic diseases, including inhalant and food allergies, in children has markedly increased to epidemic proportions [25, 29], for which the reasons are not fully clear. Childhood allergic diseases may affect the quality of life, because children with an allergic disease receive more medication and visit a family physician or hospital more frequently than children without an allergic disease [24]. Furthermore, allergic diseases have great social and economic impact both on individuals and their family members [25]. A recent register-based study in Danish and Swedish children showed that the lifetime prevalence at age 10 years of asthma and allergic rhinitis was 15.6% and 20.4%, respectively [15].

Recently, results from the PASTURE cohort study showed a distinct group of children with severe atopy characterized by high specific immunoglobulin E (IgE) levels and a strong propensity for asthma, hay fever, eczema and impaired lung function [16]. Furthermore, a recent review on European children until age 17 reported a prevalence of symptoms combined with allergic sensitization to at least one allergen of 3.6% [23].

Factors associated with the development of allergic diseases are not yet fully understood, but parental pet keeping and history of atopy showed to have a certain effect [21, 22]. Moreover, it is suggested that most children with allergic diseases start to have symptoms in early life and that early-life influences are critically important in the development of allergic diseases [1].

Studies on inhalant and food allergy in Dutch children are scarce. One study reported sensitization to inhalant allergens in 19.1% of 512 Dutch children [35], and another study reported food allergy, measured with questionnaires, in 7.2% of Dutch children [6].

So far, there are no large prospective cohort studies on presence and associated factors for the development of allergic sensitization and allergy in Dutch children.

We aimed to study the presence of inhalant and food allergic sensitization and physician-diagnosed inhalant and food allergy in Dutch children at age 10 years and its association with parental and child characteristics.

## Methods

### General design

The Generation R study is designed to identify early environmental and genetic causes and causal pathways leading to normal and abnormal growth, development and health from fetal life, childhood and young adulthood. Pregnant women with an expected delivery date between April 2002 and January 2006 living in Rotterdam were eligible for participation in the study. Around the ages of 10 years, all children and their parents were invited to visit our research centre in the Erasmus MC Sophia Children’s Hospital to participate in hands-on measurements, advanced imaging modalities, behavioural observations and biological sample collection. [17]. For the current study, cross-sectional information was obtained on allergic sensitization and allergy. The study has been approved by the Medical Ethical Committee of the Erasmus Medical Center, Rotterdam, the Netherlands (MEC-2012-165). Written informed consent was obtained from either parents or legal guardians.

### Allergic sensitization and allergy

At a median age of 9.8 years (2.5–97.5th percentile, 9.4–10.7), participating children visited our research centre. Inhalant and food allergic sensitization (no, yes) was measured by skin prick tests. Histamine dihydrochloride (10 mg/mL) and a saline solution (NaCl 0.9%) served as 2 positive controls and 1 negative control, respectively. Inhalant allergens comprised house dust mite (HDM) (*Dermatophagoides pteronyssinus*), 5-grass pollen mixture (*Dactylis glomerata*, *Festuca pratensis*, *Lolium perenne*, *Phleum pratense*, *Poa pratensis*), birch (BP) (*Betula verrucosa*), cat (*Felis catus*) and dog (*Canis familiaris*) (ALK-Abelló B.V., Almere, the Netherlands). Homemade food allergen extracts comprised hazelnut, cashew, peanut and peach [8, 9, 37]. Skin responses were recorded 15 min after applying allergens to the skin by measuring the area of the wheal (in mm^2^) using the Precise Automated Area Measurement of Skin Test (PAAMOST) software and considered positive if the area of the wheal was ≥ 40% of the histamine response [37].

Questions adapted from the International Study of Asthma and Allergies in Childhood (ISAAC) core questionnaires provided information on physician-diagnosed inhalant allergy (‘Was your child ever diagnosed with an allergy to pollen (hay fever)/house dust mite/cat/dog?’) (no, yes) and food allergies (‘Was your child ever diagnosed by a physician with an allergy to cashew/peanut?’) (no/yes) at age 10 years [4]. We further combined allergic sensitization and physician-diagnosed allergy into the following groups: ‘no allergic sensitization and no allergy’; ‘any allergic sensitization, but no allergy’; ‘no allergic sensitization, but any allergy’; and ‘any allergic sensitization and any allergy’. Allergic sensitization was defined as being sensitized to at least one inhalant allergen and to have inhalant allergy when diagnosed for at least one inhalant allergen, irrespective of a possible food allergic sensitization or food allergy. Similar considerations were made for food allergic sensitization and food allergy.

### Maternal, paternal and child characteristics

Information on maternal and paternal age, history of allergy, eczema or asthma (no, yes), BMI, maternal parity (nulliparous, multiparous) and pet keeping (no, yes) was obtained by postal questionnaires completed at enrolment. Information on child’s sex, gestational age at birth and birth weight was obtained from obstetric and midwife records at birth. Child’s ethnicity was based on the country of birth of the parents and categorized into Western (Dutch, other European, American and Oceanian), Turkish and Moroccan, African (Cape Verdean, other African, Surinamese-Creole and Dutch Antillean) and Asian (Indonesian, other Asian, Surinamese-Hindu and Surinamese-unspecified) as described earlier [14, 39]. We used questionnaires to obtain information on day care attendance (no, yes) at age 1 year and asthma and eczema ever (no, yes) at age 10 years.

### Statistical analysis

We compared characteristics of children included and not included using independent samples *t* tests, Mann-Whitney *U* tests and Pearson’s chi-square tests. We used logistic regression or multinomial logistic regression models to examine the associations of maternal, paternal and child characteristics with the risk of allergic sensitization and physician-diagnosed allergy or combined allergic sensitization and physician-diagnosed allergy groups, respectively, at age 10 years. Models were mutually adjusted for all characteristics. Also, analyses with inhalant or food allergic sensitization or allergy as the outcomes were mutually adjusted for each other. To reduce potential bias from missing data and to increase efficiency, we performed a multiple-imputation analysis of all characteristics generating 10 independent datasets using the Markov chain Monte Carlo method and calculated pooled estimates. The size or direction of the effect estimates did not materially differ between analyses with imputed data and complete cases only (data not shown). Therefore, we present results based on imputed analyses only. Measures of association are presented as adjusted odds ratios (aOR) with their 95% confidence intervals (CI). For association analyses, we applied Bonferroni correction to account for multiple testing, correcting for all fifteen characteristics. We considered a *P* value of smaller than 0.05/15 = 0.003 significant. Statistical analyses were performed using SPSS 21.0.0.1 (IBM Corp., Armonk, NY, USA).

## Results

### General

In total, 7393 children at age 10 years were included in the study. Twins (*n* = 185) and children without data on allergic sensitization or allergy (*n* = 1737) were excluded, leaving a total of 5471 children for the analyses (Supplementary Figure).

Maternal, paternal and child characteristics are presented in Tables 1 and 2 and Supplementary Table 1. Inhalant or food allergic sensitization was present in 32.2% (*n* = 1309) and 7.1% (*n* = 288) of the children at age 10 years, respectively. More specifically, children were most frequently sensitized to house dust mite (HDM) (20.2%; *n* = 823) for inhalant allergens and hazelnut (4.1%; *n* = 167) for food allergens. Physician-diagnosed inhalant or food allergy was present in 12.4% (*n* = 586) and 2.3% (*n* = 104) of the children at age 10 years, respectively, with HDM allergy giving the highest percentage. (8.0%; *n* = 381). Children who were not included in the study, had a lower gestational age at birth, were more often of non-European origin, attended day care more often and less often had eczema than those included in the study. Furthermore, their mothers and fathers were younger and more often of non-European origin, and their mothers had higher parity and BMI at enrolment (Supplementary Table 2).

**Table 1:** Characteristics of mothers and fathers

	*n* = 5471	
	Mother	Father
Age at enrolment (years)^#^	31.0 (4.9)	33.5 (5.6)
History of allergy, eczema or asthma, yes (%)	39.0 (2134)	34.3 (1874)
Parity, ≥ 1 (%)	42.6 (2332)	–
Pet keeping during pregnancy, yes (%)	34.5 (1888)	–
Body mass index at enrolment (kg/m^2^)^†^	23.7 (18.9–35.6)	25.0 (19.6–33.2)
Ethnic origin (%)		
Western	70.7 (3868)	
Turkish and Moroccan	10.9 (599)	
African	10.3 (561)	
Asian	8.1 (443)	

Values are ^#^means (SD), ^†^medians (2.5–97.5th percentile) or percentages (absolute numbers) and based on imputed data

**Table 2:** Characteristics of children

	*n* = 5471
Sex, female (%)	50.2 (2747)
Gestational age at birth (weeks)^†^	40.1 (35.7–42.3)
Birth weight (grams)^#^	3439 (555)
Ethnic origin (%)	
Western	70.7 (3868)
Turkish and Moroccan	10.9 (599)
African	10.3 (561)
Asian	8.1 (443)
Day care attendance until age 1 year, yes (%)	56.9 (3111)
Asthma ever at age 10 years, yes (%)	10.9 (595)
Eczema ever at age 10 years, yes (%)	23.8 (1303)
Inhalant allergic sensitization at age 10 years, yes (%)	
House dust mite	20.2 (823)
5-grass pollen	16.0 (651)
Birch pollen	10.7 (436)
Cat	10.6 (430)
Dog	4.3 (173)
Any	32.2 (1309)
Food allergic sensitization at age 10 years, yes (%)	
Hazelnut	4.1 (167)
Cashew nut	1.3 (53)
Peanut	3.2 (129)
Peach	3.8 (155)
Any	7.1 (288)
Physician-diagnosed inhalant allergy at age 10 years, yes (%)	
House dust mite	8.0 (381)
Hay fever	7.9 (373)
Cat	5.0 (237)
Dog	3.5 (164)
Any	12.4 (586)
Physician-diagnosed food allergy at age 10 years, yes (%)	
Cashew nut	1.4 (64)
Peanut	2.0 (93)
Any	2.3 (104)
Allergic sensitization and allergy combined at age 10 years (%)	
No allergic sensitization and no allergy	66.1 (2167)
Any allergic sensitization, but no allergy	22.6 (741)
No allergic sensitization, but any allergy	1.4 (46)
Any allergic sensitization and any allergy	9.8 (322)

Values are ^#^means (SD), ^†^medians (2.5–97.5th percentile) or percentages (absolute numbers) and based on imputed data. Data on allergic sensitizations and physician-diagnosed allergies were not imputed

### Allergic sensitization

Maternal and paternal history of allergy, eczema or asthma was associated with an increased risk of child’s allergic sensitization (aOR, 95% CI 1.44, 1.23–1.70 and 1.59, 1.30–1.94, respectively) (Table 3), which applied to all specific inhalant allergens(Supplementary Table 3). Pet keeping during pregnancy was only associated with a decreased risk of grass pollen sensitization (0.66, 0.52–0.83). Female children had a decreased risk of inhalant allergic sensitization (0.69, 0.59–0.80), except for dog allergic sensitization. Children with African ethnicity had an increased risk of inhalant allergic sensitization (1.47, 1.14–1.88), except cat and dog. Asthma and eczema ever at age 10 years were associated with an increased risk of almost all inhalant allergic sensitizations (1.65, 1.26–2.17 and 2.01, 1.68–2.41, respectively).

**Table 3:** Associations of maternal, paternal and child characteristics with allergic sensitizations in children at age 10 years

	Odds ratio (95% CI) for allergic sensitization	
	Any inhalant^a^ *n* = 4061	Any food^b^ *n* = 4051
Maternal characteristics		
Age at enrolment		
Per 1-unit increase	1.03 (1.00, 1.05)*	0.98 (0.93, 1.02)
History of allergy, eczema or asthma		
No	Reference	Reference
Yes	1.44 (1.23, 1.70)**	1.18 (0.84, 1.67)
Parity		
0	Reference	Reference
≥ 1	0.89 (0.75, 1.05)	1.01 (0.72, 1.43)
Pet keeping during pregnancy		
No	Reference	Reference
Yes	0.77 (0.64, 0.92)*	0.81 (0.55, 1.20)
Body mass index at enrolment		
Per 1-unit increase	1.00 (0.98, 1.02)	1.01 (0.97, 1.05)
Paternal characteristics		
Age at enrolment		
Per 1-unit increase	0.98 (0.96, 1.01)	1.02 (0.98, 1.06)
History of allergy, eczema or asthma		
No	Reference	Reference
Yes	1.59 (1.30, 1.94)**	1.19 (0.83, 1.71)
Body mass index at enrolment		
Per 1-unit increase	1.01 (0.98, 1.04)	1.00 (0.94, 1.07)
Child characteristics		
Sex		
Male	Reference	Reference
Female	0.69 (0.59, 0.80)**	1.13 (0.82, 1.55)
Gestational age at birth		
Per 1-unit increase	1.03 (0.98, 1.09)	0.98 (0.87, 1.09)
Birth weight		
Per 500-unit increase	0.97 (0.89, 1.06)	1.00 (0.83, 1.21)
Ethnic origin		
Western	Reference	Reference
Turkish and Moroccan	1.05 (0.80, 1.37)	1.06 (0.59, 1.88)
African	1.47 (1.14, 1.88)**	1.09 (0.67, 1.77)
Asian	1.15 (0.87, 1.53)	1.09 (0.63, 1.91)
Day care attendance until age 1 year		
No	Reference	Reference
Yes	0.90 (0.71, 1.13)	0.94 (0.62, 1.41)
Asthma ever at age 10 years		
No	Reference	Reference
Yes	1.65 (1.26, 2.17)**	1.13 (0.68, 1.87)
Eczema ever at age 10 years		
No	Reference	Reference
Yes	2.01 (1.68, 2.41)**	2.09 (1.44, 3.02)**

Values are odds ratios (95% confidence interval) from logistic regression models based on imputed data. Models are adjusted for all characteristics. ^a^Additionally adjusted for food allergic sensitization. ^b^Additionally adjusted for inhalant allergic sensitization. *P value < 0.05. **P value < 0.003

For the outcome of food allergic sensitization, only eczema ever at age 10 years was consistently associated with an increased risk of sensitization to any food allergen (2.09, 1.44–3.02) (Table 3), specifically for cashew (2.93, 1.49–5.75), peanut (2.22, 1.36–3.62) and peach (1.77, 1.09–2.88) (Supplementary Table 4). No consistent associations of other characteristics with allergic sensitization were found.

### Allergy

Maternal or paternal history of allergy, eczema or asthma was associated with increased risks of physician-diagnosed inhalant allergy (1.43, 1.15–1.78 and 1.69, 1.31–2.18, respectively) (Table 4), which specifically comprised almost all inhalant allergies (Supplementary Table 4). Asthma ever and eczema ever at age 10 years were associated with an increased risk of physician-diagnosed inhalant allergy (4.60, 3.55–5.96 and 2.42, 1.94–3.03, respectively), with the highest risk of HDM allergy (6.06, 4.53–8.10) and dog allergy (5.95, 3.83–9.24) (Supplementary Table 5).

**Table 4:** Associations of maternal, paternal and child characteristics with physician-diagnosed allergies in children at age 10 years

	Odds ratio (95% CI) for physician-diagnosed allergy	
	Any inhalant^a^ *n* = 4725	Any food^b^ *n* = 4621
Maternal characteristics		
Age at enrolment		
Per 1-unit increase	0.97 (0.94, 1.01)	1.03 (0.96, 1.11)
History of allergy, eczema or asthma		
No	Reference	Reference
Yes	1.43 (1.15, 1.78)**	1.01 (0.60, 1.71)
Parity		
0	Reference	Reference
≥ 1	0.83 (0.65, 1.05)	0.93 (0.55, 1.59)
Pet keeping during pregnancy		
No	Reference	Reference
Yes	0.82 (0.64, 1.05)	0.92 (0.52, 1.62)
Body mass index at enrolment		
Per 1-unit increase	1.00 (0.97, 1.03)	0.98 (0.92, 1.05)
Paternal characteristics		
Age at enrolment		
Per 1-unit increase	1.01 (0.98, 1.04)	1.00 (0.93, 1.06)
History of allergy, eczema or asthma		
No	Reference	Reference
Yes	1.69 (1.31, 2.18)**	1.50 (0.83, 2.68)
Body mass index at enrolment		
Per 1-unit increase	1.00 (0.96, 1.05)	0.96 (0.87, 1.06)
Child characteristics		
Sex		
Male	Reference	Reference
Female	0.68 (0.55, 0.84)**	1.12 (0.68, 1.83)
Gestational age at birth		
Per 1-unit increase	0.99 (0.92, 1.06)	0.96 (0.81, 1.13)
Birth weight		
Per 500-unit increase	1.02 (0.90, 1.15)	0.97 (0.73, 1.28)
Ethnic origin		
Western	Reference	Reference
Turkish and Moroccan	1.50 (1.02, 2.21)*	0.45 (0.14, 1.53)
African	1.39 (0.98, 1.97)	1.67 (0.82, 3.43)
Asian	1.40 (0.95, 2.06)	1.97 (0.89, 4.35)
Day care attendance until age 1 year		
No	Reference	Reference
Yes	1.03 (0.72, 1.47)	0.84 (0.41, 1.74)
Asthma ever at age 10 years		
No	Reference	Reference
Yes	4.60 (3.55, 5.96)**	1.70 (0.89, 3.24)
Eczema ever at age 10 years		
No	Reference	Reference
Yes	2.42 (1.94, 3.03)**	5.38 (3.04, 9.52)**

Values are odds ratios (95% confidence interval) from logistic regression models based on imputed data. Models are adjusted for all characteristics. ^a^Additionally adjusted for physician-diagnosed food allergy. ^b^Additionally adjusted for physician-diagnosed inhalant allergy. *P value < 0.05. **P value < 0.003

Only eczema ever at age 10 years was consistently associated with an increased risk of physician-diagnosed food allergy (5.38, 3.04–9.52), with the highest risk for cashew (7.36, 3.20–16.94) and peanut (5.58, 3.08–10.10) (Supplementary Table 6). No associations of other characteristics with physician-diagnosed allergy to any allergen in the children were found.

### Allergic sensitization and physician-diagnosed allergy combined

Maternal and paternal history of allergy, eczema or asthma was associated with increased risks of children with ‘any allergic sensitization and no physician diagnosed allergy’ (1.44, 1.19–1.74 and 1.65, 1.34–2.04 for mothers and fathers, respectively) and with ‘any allergic sensitization and any physician diagnosed allergy’ (1.73, 1.31–2.28 and 2.07, 1.56–2.75 for mother and fathers, respectively), compared with no parental history of allergy, eczema or allergy and no allergic sensitization and physician-diagnosed allergy (Table 5). Being a child with African ethnicity was consistently associated with all three combinations, with highest OR for ‘no allergic sensitization but any allergy’ (4.99, 2.24–11.1) Asthma ever at 10 years was associated with increased risk of children with ‘any allergic sensitization and any physician diagnosed allergy’ (5.14, 3.65–7.23). Eczema ever at 10 years of age was consistently associated with all three combinations, with highest OR for children with ‘any allergic sensitization and any physician-diagnosed allergy’ (3.96, 2.95–5.31). Parity was associated with ‘any allergic sensitization, without allergy’ (0.78, 0.64–0.96). No consistent associations of other characteristics with combined allergic sensitization and physician-diagnosed allergy were found.

**Table 5:** Associations of maternal, paternal and child characteristics with allergic sensitization and physician-diagnosed allergy combined in children at age 10 years

	Odds ratio (95% CI) for any allergic sensitization and any physician-diagnosed allergy combined		
	Any allergic sensitization, but no allergy *n* = 741	No allergic sensitization, but any allergy *n* = 46	Any allergic sensitization and any allergy *n* = 322
Maternal characteristics			
Age at enrolment			
Per 1-unit increase	1.03 (1.00, 1.06)	0.95 (0.88, 1.03)	0.99 (0.95, 1.03)
History of allergy, eczema or asthma			
No	Reference	Reference	Reference
Yes	1.44 (1.19, 1.74)**	1.18 (0.57, 2.47)	1.73 (1.31, 2.28)**
Parity			
0	Reference	Reference	Reference
≥ 1	0.83 (0.69, 1.01)*	0.81 (0.41, 1.62)	0.83 (0.62, 1.12)
Pet keeping during pregnancy			
No	Reference	Reference	Reference
Yes	0.78 (0.64, 0.96)*	0.96 (0.46, 2.03)	0.74 (0.53, 1.02)
Body mass index at enrolment			
Per 1-unit increase	1.00 (0.98, 1.03)	1.04 (0.97, 1.11)	0.97 (0.93, 1.01)
Paternal characteristics			
Age at enrolment			
Per 1-unit increase	0.98 (0.96, 1.00)	1.01 (0.93, 1.08)	1.01 (0.97, 1.05)
History of allergy, eczema or asthma			
No	Reference	Reference	Reference
Yes	1.65 (1.34, 2.04)**	1.44 (0.61, 3.43)	2.07 (1.56, 2.75)**
Body mass index at enrolment			
Per 1-unit increase	1.01 (0.98, 1.04)	0.97 (0.87, 1.09)	1.00 (0.95, 1.06)
Child characteristics			
Sex			
Male	Reference	Reference	Reference
Female	0.71 (0.59, 0.84)**	0.68 (0.37, 1.26)	0.62 (0.47, 0.80)**
Gestational age at birth			
Per 1-unit increase	1.02 (0.96, 1.08)	0.93 (0.76, 1.13)	1.03 (0.94, 1.14)
Birth weight			
Per 500-unit increase	0.99 (0.89, 1.09)	1.01 (0.71, 1.44)	0.99 (0.85, 1.15)
Ethnic origin			
Western	Reference	Reference	Reference
Turkish and Moroccan	1.00 (0.70, 1.42)	1.69 (0.58, 4.94)	1.43 (0.88, 2.34)
African	1.69 (1.25, 2.30)**	4.99 (2.24, 11.1)**	1.88 (1.22, 2.90)*
Asian	1.34 (0.95, 1.88)	3.08 (1.18, 8.06)*	1.61 (1.01, 2.57)*
Day care attendance until age 1 year			
No	Reference	Reference	Reference
Yes	0.81 (0.64, 1.03)	0.53 (0.23, 1.21)	0.86 (0.58, 1.28)
Asthma ever at age 10 years			
No	Reference	Reference	Reference
Yes	0.85 (0.59, 1.21)	3.07 (1.45, 6.50)*	5.14 (3.65, 7.23)**
Eczema ever at age 10 years			
No	Reference	Reference	Reference
Yes	2.37 (1.93, 2.91)**	3.25 (1.69, 6.23)**	3.96 (2.95, 5.31)**

Values are odds ratios (95% confidence interval) from multinomial logistic regression models based on imputed data. Reference group is children without any allergic sensitization or physician-diagnosed allergy (*n* = 2167). Models are adjusted for all characteristics. *P value < 0.05. **P value < 0.003

## Discussion

This large study showed high presences of inhalant and food allergic sensitizations and physician-diagnosed allergies in this group of Dutch children at age 10 years. Maternal and paternal history of allergy, asthma and eczema and child’s current asthma and eczema were most consistently associated with increased risks of allergic sensitization to inhalant and food allergens, physician-diagnosed allergies or combined groups, while female sex of the child was associated with decreased risks of some of these outcomes.

The percentage of sensitization to inhalant sensitization was 32% in our study. Earlier reports on inhalant sensitization in children in large cross-sectional studies found a prevalence rate of approximately 40% [23, 29]. The prevalence of physician-diagnosed allergy to HDM, hay fever and any pet in the current study was 8.0%, 7.9% and 12.4%, respectively. Higher prevalence for allergy to HDM and grass pollen was found in a recent Polish study among children at age 7 years (13.5% and 11.8%, respectively) [34]. In this group of children aged 10 years, we also found that girls were consistently less likely than boys to develop sensitization to inhalant allergens, as well as physician-diagnosed inhalant allergy at the age of 10 years (OR 0.69 and 0.68). This agrees with the findings of previous European studies [5, 30].

Furthermore, the percentage of sensitization to food allergens including hazelnut, cashew, peanut and peach was 4.1%, 1.3%, 3.2% and 3.8%, respectively, in our study. Previous studies in Europe showed similar prevalence [26], including sensitization to food allergens (all kind of allergens) of 4.1% in children at age 10 years (*n* = 969) in a birth cohort study on the Isle of Wight (UK) [38].

Allergic sensitization to food allergens occurs often without clinical relevance, and therefore, physician-diagnosed food allergy is of special interest. The presence of physician-diagnosed allergy to cashew, peanut or any food was 1.4%, 2.0% and 2.3%, respectively, in our study, comparable with recent results on prevalence of tree nut allergy in Europe by McWilliam et al. (0.2–4.9%) [19].

We found several associations between history of allergy in the parents and sensitization or physician-diagnosed allergy in the children. A history of allergy, eczema or asthma of the parents showed associations with increased risk of sensitization to inhalant allergens and doctor-diagnosed inhalant allergy in children at age 10 years. These parental effects on inhalant allergy in children are well known in literature [2, 5].

We demonstrated that pet exposure during pregnancy was negatively associated only with inhalant sensitization in children at age 10 years but significance disappeared after Bonferroni correction. It has previously been demonstrated that pet avoidance is associated with specific IgE responses and increased specific IgE levels [29, 37]. Furthermore, several prospective studies indicated that living with a pet reduces the risk of becoming sensitized to that pet [33], while other studies did not [13, 32]. Therefore, the relationship between the development of sensitization and exposure to pet allergens remains controversial.

Neither history of the parents nor pet keeping during pregnancy nor day care of the child until age 1 was associated with sensitization to food allergens or physician-diagnosed food allergy, while eczema ever at age 10 years had a large effect on sensitization to the tested food allergens (hazelnut, cashew, peanut and peach) and physician-diagnosed food allergy at 10 years.

Asthma and eczema ever in children in the current study at age 10 were strongly associated with physician-diagnosed allergy to inhalant allergens. This is in line with a study by Arshad et al. in a birth cohort of 1456 children, reporting that 80% of the children at age 4 years, with positive skin test to more than 4 allergens, had asthma, eczema and/or rhinitis compared with 20% of non-atopic children [3].

Atopic dermatitis seems to play an important role in the development of food allergy. Recent findings in the HealthNuts study found 26% of the children at age 2 years with atopic dermatitis had food allergy [27]. In the current study, high ORs were found for having ever eczema and physician-diagnosed cashew allergy (OR (95% CI), 7.87 (3.45–17.92)). In an earlier study in the Netherlands, van der Valk et al. found a high number of positive challenges in cashew-sensitized children (76.4%) [36]. Apparently, cashew is a common allergen in the Netherlands and becomes symptomatic especially in combination with eczema at a young age.

We found an association between children with African ethnicity and sensitization to inhalant allergens. Some other studies found that ethnicity is a risk factor for the development of sensitization to foods [29] and inhalant allergens [18], but this might be explained rather by environmental than genetic factors [40].

The major strength of this study is the use of a population-based prospective study design from fetal life onwards with a large number of participants and detailed information on parental and child characteristics and allergic sensitization and allergy. We were able to study multiple potential associated maternal, paternal and child characteristics. Previously, we observed no associations of duration and exclusiveness of breastfeeding with any allergic sensitization or allergy, or combination of these outcomes at age 10 years, and therefore not included breastfeeding in our models [11]. Furthermore, in the same population, timing and introduction of allergenic foods, e.g. cow’s milk, were studied, and no effects were seen in allergic sensitization and physician-diagnosed allergy, so these outcomes were also not included in our model [10].

Some methodological limitations should be considered. First, selection bias in longitudinal studies mainly arises due to follow-up. Characteristics of non-included subjects differed from those included in the study. Firstly, this may affect the percentage of estimated prevalence of allergic sensitization and physician-diagnosed allergy in the study. The children in the non-included group had a slightly shorter gestational age at birth. The latter is earlier described as being negatively associated with food allergy in children [20]. Furthermore, the included group had a higher percentage (not significant) of asthma and eczema ever at age 10 compared with the non-included group. The included group might be somewhat more atopic and consequently might show higher numbers for allergic sensitization. Although the differences between included and non-included children may affect the generalizability of our results, it is unlikely that these differences led to significant selection bias in this study. Second, we used parental questionnaires to obtain data on child’s allergy and eczema, which may have led to non-differential misclassification of these outcomes, specifically food allergy. This probably led to an underestimation of the effect estimates. However, questionnaires consisted of commonly used questions that reflect the prevalence of allergy in young children at the population level [4, 12]. Third, we aimed to reduce potential bias due to missing data using multiple imputation methods assuming that missing data were missing at random. However, it remains difficult to quantify the effect of non-random missing data, partly due to computational limitations and current statistical software availability. Fourth, for measuring allergic sensitization, we selected a panel of common inhalant and food allergens relevant to children of age 10 years. Apparently, we selected allergens that were relevant for children of this age, with high sensitization levels. Milk and egg allergens were not considered because of low sensitization and allergy rates at this age [28]. Finally, as in any observational study, residual confounding due to insufficiently or unmeasured confounders might be an issue.

Although progress has been made in defining endotypes for asthma, definitions for food and inhalant allergy lag behind. Recently, Muraro et al. suggested to combine all current known predictive markers to understand and differentiate the distinct endotypes of disease among individuals having a similar phenotype and assist with the ability to personalize treatment and prevention to improve health outcome [21, 22]. The current study underlines the relation between several known predictive markers. Furthermore, development of allergy in children is often called ‘the atopic march’. The results in this cohort study on children from birth until age 10 years confirm the strong relation between eczema, asthma and inhalant and food allergy, which often starts with atopic dermatitis at a very young age [7]. Early recognition of atopic dermatitis and consequent treatment might have effect on further development of atopic diseases [31].

In conclusion, we described the results of a large population-based cohort study on the presence of allergic sensitization and physician-diagnosed allergy in Dutch children at age 10 years. We found strong effect estimates for the associations of parental history of allergy, eczema or asthma with physician-diagnosed inhalant allergy. Also, eczema ever at age 10 years was a risk factor for the development of physician-diagnosed inhalant and food allergy, and therefore, for children with eczema, a proper and intense guidance by paediatricians is mandatory. Further studies using more detailed information on allergic sensitization and allergy at different time points form early-life onwards are needed to provide better insights in the underlying biological mechanisms.

## Electronic supplementary material

Figure S1 (DOCX 25 kb)

Table S1 (DOCX 16 kb)

Table S2 (DOCX 17 kb)

Table S3 (DOCX 17 kb)

Table S4 (DOCX 17 kb)

Table S5 (DOCX 17 kb)

Table S6 (DOCX 16 kb)

## Abbreviations

aOR: Adjusted odds ratio
BMI: Body mass index
CI: Confidence interval
HDM: House dust mite
IgE: Immunoglobulin E
