# Supplementary material for: Parental and child factors associated with inhalant and food allergy in a population-based prospective cohort study: the Generation R Study
Source: Eur J Pediatr. 2019 Aug 15;178(10):1507–17. doi: 10.1007/s00431-019-03441-5 (PMC6733817; doi:10.1007/s00431-019-03441-5)
Supplement: Supplementary file 1 — (DOCX 25 kb) [file 431_2019_3441_MOESM1_ESM.docx]

**Supplementary Figure.** Flowchart of participants.

**n = 7,393**

Children with participation at age 10 years

**n = 7,208**

Singleton children

**n = 5,471**

Children with any data on eczema, allergic sensitization or allergy available

Allergic sensitization at age 10 years

Any inhalant n = 4,061

Any food n = 4,051

Physician-diagnosed allergy

at age 10 years

Any inhalant n = 4,725

Any food n = 4,621

**n = 1,737**

Children without data on allergic sensitization or allergy excluded

**n = 185**

Twins excluded
