# Supplementary material for: Parental and child factors associated with inhalant and food allergy in a population-based prospective cohort study: the Generation R Study
Source: Eur J Pediatr. 2019 Aug 15;178(10):1507–17. doi: 10.1007/s00431-019-03441-5 (PMC6733817; doi:10.1007/s00431-019-03441-5)
Supplement: Supplementary file 2 — (DOCX 16 kb) [file 431_2019_3441_MOESM2_ESM.docx]

**Supplementary Table 1.** Characteristics of mothers, fathers and children (n = 5,471).

|  | **Observed** | **Imputed** |
| --- | --- | --- |
| **Maternal characteristics** |  |  |
| Age at enrolment (years)^#^ | 31.0 (4.9) | 31.0 (4.9) |
| *Missing* | *0 (0)* | *0 (0)* |
| History of allergy, eczema or asthma (%) |  |  |
| No | 60.5 (2,687) | 61.0 (3,337) |
| Yes | 39.5 (1,752) | 39.0 (2,134) |
| *Missing* | *18.9 (1,032)* | *0 (0)* |
| Parity (%) |  |  |
| 0 | 57.6 (3,050) | 57.4 (3,139) |
| ≥1 | 42.4 (2,247) | 42.6 (2,332) |
| *Missing* | *3.2 (174)* | *0 (0)* |
| Pet keeping during pregnancy (%) |  |  |
| No | 66.2 (2,841) | 65.5 (3,583) |
| Yes | 33.8 (1,450) | 34.5 (1,888) |
| *Missing* | *21.6 (1,180)* | *0 (0)* |
| Body mass index at enrolment (kg/m^2^)^†^ | 23.7 (18.8-35.5) | 23.7 (18.9-35.6) |
| *Missing* | *10.1 (552)* | *0 (0)* |
| **Paternal characteristics** |  |  |
| Age at enrolment (years)^#^ | 33.4 (5.4) | 33.5 (5.6) |
| *Missing* | *28.7 (1,570)* | *0 (0)* |
| History of allergy, eczema or asthma (%) |  |  |
| No | 65.9 (2,320) | 65.7 (3,597) |
| Yes | 34.1 (1,200) | 34.3 (1,874) |
| *Missing* | *35.7 (1,951)* | *0 (0)* |
| Body mass index at enrolment (kg/m^2^)^†^ | 24.9 (19.6-32.9) | 25.0 (19.6-33.2) |
| *Missing* | *28.8 (1,577)* | *0 (0)* |
| **Child characteristics** |  |  |
| Sex (%) |  |  |
| Male | 49.8 (2,724) | 49.8 (2,724) |
| Female | 50.2 (2,747) | 50.2 (2,747) |
| *Missing* | *0 (0)* | *0 (0)* |
| Gestational age at birth (weeks)^†^ | 40.1 (35.7-42.3) | 40.1 (35.7-42.3) |
| *Missing* | *0.6 (33)* | *0 (0)* |
| Birth weight (grams)^#^ | 3,439 (554) | 3,439 (555) |
| *Missing* | *0.1 (7)* | *0 (0)* |
| Ethnic origin (%) |  |  |
| Western | 71.5 (3,830) | 70.7 (3,868) |
| Turkish and Moroccan | 10.9 (582) | 10.9 (599) |
| African | 10.1 (542) | 10.3 (561) |
| Asian | 7.6 (405) | 8.1 (443) |
| *Missing* | *2.0 (112)* | *0 (0)* |
| Day care attendance until age 1 year (%) |  |  |
| No | 36.9 (1,241) | 43.1 (2,360) |
| Yes | 63.1 (2,119) | 56.9 (3,111) |
| *Missing* | *38.6 (2,111)* | *0 (0)* |
| Asthma ever at age 10 years (%) |  |  |
| No | 90.5 (4,243) | 89.1 (4,876) |
| Yes | 9.5 (447) | 10.9 (595) |
| *Missing* | *14.3 (781)* | *0 (0)* |
| Eczema ever at age 10 years (%) |  |  |
| No | 77.2 (3,612) | 76.2 (4,168) |
| Yes | 22.8 (1,064) | 23.8 (1,303) |
| *Missing* | *14.5 (795)* | *0 (0)* |

Values are ^#^means (SD), ^†^medians (2.5-97.5th percentile) or percentages (absolute numbers) and based on observed and imputed data. Data on allergic sensitizations and physician-diagnosed allergies were not imputed.
