# Supplementary material for: Parental and child factors associated with inhalant and food allergy in a population-based prospective cohort study: the Generation R Study
Source: Eur J Pediatr. 2019 Aug 15;178(10):1507–17. doi: 10.1007/s00431-019-03441-5 (PMC6733817; doi:10.1007/s00431-019-03441-5)
Supplement: Supplementary file 3 — (DOCX 17 kb) [file 431_2019_3441_MOESM3_ESM.docx]

**Supplementary Table 2.** Characteristics of mothers, fathers and children included and not included in the study.

|  | **Included**  **n = 5,471** | **Not included**  **n = 1,737** | **P-value for difference** |
| --- | --- | --- | --- |
| **Maternal characteristics** |  |  |  |
| Age at enrolment (years)^#^ | 31.0 (4.9) | 29.0 (5.4) | **<0.001*** |
| *Missing* | *0 (0)* | *0.1 (1)* |  |
| History of allergy, eczema or asthma (%) |  |  | 0.23 |
| No | 60.5 (2,687) | 62.4 (813) |  |
| Yes | 39.5 (1,752) | 37.6 (490) |  |
| *Missing* | *18.9 (1,032)* | *25.0 (434)* |  |
| Parity (%) |  |  | **<0.001*** |
| 0 | 57.6 (3,050) | 50.8 (853) |  |
| ≥1 | 42.4 (2,247) | 49.2 (827) |  |
| *Missing* | *3.2 (174)* | *3.3 (57)* |  |
| Pet keeping during pregnancy (%) |  |  | 0.50 |
| No | 66.2 (2,841) | 67.2 (863) |  |
| Yes | 33.8 (1,450) | 32.8 (421) |  |
| *Missing* | *21.6 (1,180)* | *26.1 (453)* |  |
| Body mass index at enrolment (kg/m^2^)^†^ | 23.7 (18.8-35.5) | 24.2 (18.4-37.0) | **<0.001*** |
| *Missing* | *10.1 (552)* | *9.7 (169)* |  |
| **Paternal characteristics** |  |  |  |
| Age at enrolment (years)^#^ | 33.4 (5.4) | 32.3 (5.9) | **<0.001*** |
| *Missing* | *28.7 (1,570)* | *40.0 (695)* |  |
| History of allergy, eczema or asthma (%) |  |  | 0.27 |
| No | 65.9 (2,320) | 67.9 (563) |  |
| Yes | 34.1 (1,200) | 32.1 (266) |  |
| *Missing* | *35.7 (1,951)* | *52.3 (908)* |  |
| Body mass index at enrolment (kg/m^2^)^†^ | 24.9 (19.6-32.9) | 25.2 (19.4-33.9) | 0.26 |
| *Missing* | *28.8 (1,577)* | *40.4 (701)* |  |
| **Child characteristics** |  |  |  |
| Sex (%) |  |  | 0.21 |
| Male | 49.8 (2,724) | 51.5 (894) |  |
| Female | 50.2 (2,747) | 48.5 (842) |  |
| *Missing* | *0 (0)* | *0.1 (1)* |  |
| Gestational age at birth (weeks)^†^ | 40.1 (35.7-42.3) | 40.0 (36.0-42.3) | **0.04*** |
| *Missing* | *0.6 (33)* | *1.0 (17)* |  |
| Birth weight (grams)^#^ | 3,439 (554) | 3,409 (554) | 0.05 |
| *Missing* | *0.1 (7)* | *0.5 (8)* |  |
| Ethnic origin (%) |  |  | **<0.001*** |
| Western | 71.5 (3,830) | 56.9 (914) |  |
| Turkish and Moroccan | 10.9 (582) | 20.9 (335) |  |
| African | 10.1 (542) | 13.8 (222) |  |
| Asian | 7.6 (405) | 8.4 (135) |  |
| *Missing* | *2.0 (112)* | *7.5 (131)* |  |
| Day care attendance until age 1 year (%) |  |  | **<0.001*** |
| No | 36.9 (1,241) | 51.6 (345) |  |
| Yes | 63.1 (2,119) | 48.4 (323) |  |
| *Missing* | *38.6 (2,111)* | *61.5 (1,069)* |  |
| Asthma ever at age 10 years (%) |  |  | 0.26 |
| No | 90.5 (4,243) | 82.4 (14) |  |
| Yes | 9.5 (447) | 17.6 (3) |  |
| *Missing* | *14.3 (781)* | *99.0 (1,720)* |  |
| Eczema ever at age 10 years (%) |  |  | 0.05 |
| No | 77.2 (3,612) | 56.3 (9) |  |
| Yes | 22.8 (1,064) | 43.8 (7) |  |
| *Missing* | *14.5 (795)* | *99.1 (1,721)* |  |

Values are *means (SD), ^†^medians (2.5-97.5th percentile) or percentages (absolute numbers) and based on observed data. P-values for difference are calculated by independent samples T-test for continuous variables with a normal distribution, the Mann-Whitney U-test for continuous variables with a skewed distribution, and Pearson's Chi-square test for categorical variables. *P-value <0.05.
