# Supplementary material for: Parental and child factors associated with inhalant and food allergy in a population-based prospective cohort study: the Generation R Study
Source: Eur J Pediatr. 2019 Aug 15;178(10):1507–17. doi: 10.1007/s00431-019-03441-5 (PMC6733817; doi:10.1007/s00431-019-03441-5)
Supplement: Supplementary file 4 — (DOCX 17 kb) [file 431_2019_3441_MOESM4_ESM.docx]

**Supplementary Table 3.** Associations of maternal, paternal and child characteristics with specific inhalant allergic sensitizations in children at age 10 years.

|  | **Odds ratio (95% CI) for specific inhalant allergic sensitization** | | | | |
| --- | --- | --- | --- | --- | --- |
|  | **House dust mite^1^**  **n = 4,067** | **5-Grass pollen^1^**  **n = 4,067** | **Birch pollen^1^**  **n = 4,066** | **Cat^1^**  **n = 4,060** | **Dog^1^**  **n = 4,066** |
| **Maternal characteristics** |  |  |  |  |  |
| Age at enrolment |  |  |  |  |  |
| Per 1-unit increase | 1.01 (0.99, 1.04) | 1.01 (0.98, 1.04) | 1.02 (0.98, 1.06) | **1.06 (1.03, 1.10)*** | 1.03 (0.98, 1.08) |
| History of allergy, eczema or asthma |  |  |  |  |  |
| No | Reference | Reference | Reference | Reference | Reference |
| Yes | **1.29 (1.08, 1.54)*** | **1.58 (1.29, 1.94)**** | **1.40 (1.07, 1.83)*** | **1.58 (1.25, 1.98)**** | 1.10 (0.78, 1.56) |
| Parity |  |  |  |  |  |
| 0 | Reference | Reference | Reference | Reference | Reference |
| ≥1 | 1.04 (0.87, 1.25) | 0.81 (0.65, 1.00) | **0.72 (0.54, 0.96)*** | 0.84 (0.65, 1.08) | **0.62 (0.42, 0.91)*** |
| Pet keeping during pregnancy |  |  |  |  |  |
| No | Reference | Reference | Reference | Reference | Reference |
| Yes | 0.90 (0.72, 1.11) | **0.66 (0.52, 0.83)**** | **0.72 (0.53, 0.98)*** | 0.77 (0.57, 1.02) | 0.97 (0.64, 1.47) |
| Body mass index at enrolment |  |  |  |  |  |
| Per 1-unit increase | 1.00 (0.97, 1.02) | 1.01 (0.99, 1.04) | 1.00 (0.97, 1.03) | **0.97 (0.94, 1.00)*** | 1.00 (0.96, 1.05) |
| **Paternal characteristics** |  |  |  |  |  |
| Age at enrolment |  |  |  |  |  |
| Per 1-unit increase | 0.99 (0.97, 1.02) | 0.98 (0.96, 1.01) | 0.99 (0.96, 1.02) | **0.97 (0.94, 1.00)*** | 0.99 (0.94, 1.03) |
| History of allergy, eczema or asthma |  |  |  |  |  |
| No | Reference | Reference | Reference | Reference | Reference |
| Yes | **1.57 (1.29, 1.91)**** | **1.42 (1.08, 1.87)*** | **1.67 (1.18, 2.36)*** | **1.85 (1.42, 2.41)**** | **1.55 (1.02, 2.35)*** |
| Body mass index at enrolment |  |  |  |  |  |
| Per 1-unit increase | 1.00 (0.98, 1.03) | 1.03 (1.00, 1.06) | 1.00 (0.95, 1.04) | 0.99 (0.96, 1.03) | 1.00 (0.96, 1.05) |
| **Child characteristics** |  |  |  |  |  |
| Sex |  |  |  |  |  |
| Male | Reference | Reference | Reference | Reference | Reference |
| Female | **0.78 (0.66, 0.92)*** | **0.78 (0.65, 0.95)** | **0.72 (0.55, 0.93)** | **0.64 (0.51, 0.81)**** | 0.78 (0.55, 1.09) |
| Gestational age at birth |  |  |  |  |  |
| Per 1-unit increase | 1.06 (1.00, 1.12) | 0.99 (0.92, 1.05) | 1.06 (0.97, 1.16) | 1.00 (0.93, 1.08) | 0.97 (0.87, 1.08) |
| Birth weight |  |  |  |  |  |
| Per 500-unit increase | 0.98 (0.89, 1.07) | 1.00 (0.89, 1.12) | 0.96 (0.83, 1.11) | 1.01 (0.89, 1.15) | 0.99 (0.81, 1.20) |
| Ethnic origin |  |  |  |  |  |
| Western | Reference | Reference | Reference | Reference | Reference |
| Turkish and Moroccan | **1.49 (1.12, 1.99)*** | 0.84 (0.60, 1.18) | 0.80 (0.49, 1.29) | 1.19 (0.79, 1.80) | 1.18 (0.65, 2.13) |
| African | **1.39 (1.06, 1.81)*** | **1.39 (1.03, 1.88)*** | **1.50 (1.02, 2.21)*** | 0.90 (0.61, 1.32) | 1.23 (0.73, 2.08) |
| Asian | 1.03 (0.75, 1.41) | 1.25 (0.90, 1.75) | 1.16 (0.74, 1.84) | 1.24 (0.81, 1.90) | 1.27 (0.71, 2.29) |
| Day care attendance until age 1 year |  |  |  |  |  |
| No | Reference | Reference | Reference | Reference | Reference |
| Yes | 1.03 (0.81, 1.32) | 0.81 (0.64, 1.02) | 0.75 (0.53, 1.05) | 1.05 (0.79, 1.40) | 0.94 (0.60, 1.47) |
| Asthma ever at age 10 years |  |  |  |  |  |
| No | Reference | Reference | Reference | Reference | Reference |
| Yes | **2.17 (1.68, 2.81)**** | 1.29 (0.96, 1.72) | 1.39 (0.89, 2.18) | **1.71 (1.21, 2.42)**** | **1.89 (1.15, 3.09)*** |
| Eczema ever at age 10 years |  |  |  |  |  |
| No | Reference | Reference | Reference | Reference | Reference |
| Yes | **1.63 (1.34, 1.99)**** | **1.88 (1.51, 2.32)**** | **2.34 (1.75, 3.13)**** | **1.90 (1.46, 2.46)**** | **1.80 (1.18, 2.74)*** |

Values are odds ratios (95% confidence interval) from logistic regression models based on imputed data. Models are adjusted for all characteristics. **^1^**Additionally adjusted for food allergic sensitization. *P-value <0.05. **P-value <0.003.
