# Supplementary material for: Parental and child factors associated with inhalant and food allergy in a population-based prospective cohort study: the Generation R Study
Source: Eur J Pediatr. 2019 Aug 15;178(10):1507–17. doi: 10.1007/s00431-019-03441-5 (PMC6733817; doi:10.1007/s00431-019-03441-5)
Supplement: Supplementary file 5 — (DOCX 17 kb) [file 431_2019_3441_MOESM5_ESM.docx]

**Supplementary Table 4.** Associations of maternal, paternal and child characteristics with specific food allergic sensitizations in children at age 10 years.

|  | **Odds ratio (95% CI) for specific food allergic sensitization** | | | |
| --- | --- | --- | --- | --- |
|  | **Hazelnut^1^**  **n = 4,057** | **Cashew nut^1^**  **n = 4,056** | **Peanut^1^**  **n = 4,058** | **Peach^1^**  **n = 4,066** |
| **Maternal characteristics** |  |  |  |  |
| Age at enrolment |  |  |  |  |
| Per 1-unit increase | 0.96 (0.90, 1.01) | **1.09 (1.00, 1.19)*** | **0.94 (0.89, 1.00)*** | 0.96 (0.90, 1.01) |
| History of allergy, eczema or asthma |  |  |  |  |
| No | Reference | Reference | Reference | Reference |
| Yes | 1.00 (0.66, 1.52) | 1.49 (0.77, 2.88) | 1.04 (0.66, 1.63) | 0.94 (0.62, 1.44) |
| Parity |  |  |  |  |
| 0 | Reference | Reference | Reference | Reference |
| ≥1 | 1.37 (0.90, 2.10) | **0.44 (0.22, 0.89)*** | 1.40 (0.88, 2.22) | 1.22 (0.78, 1.92) |
| Pet keeping during pregnancy |  |  |  |  |
| No | Reference | Reference | Reference | Reference |
| Yes | 0.81 (0.49, 1.34) | 1.07 (0.55, 2.10) | 1.26 (0.71, 2.24) | 0.81 (0.49, 1.34) |
| Body mass index at enrolments |  |  |  |  |
| Per 1-unit increase | 1.02 (0.98, 1.06) | 0.96 (0.88, 1.04) | 0.99 (0.93, 1.05) | 1.01 (0.96, 1.06) |
| **Paternal characteristics** |  |  |  |  |
| Age at enrolments |  |  |  |  |
| Per 1-unit increase | 1.04 (0.99, 1.09) | 1.04 (0.55, 1.99) | 1.05 (1.00, 1.10) | 1.04 (0.99, 1.10) |
| History of allergy, eczema or asthma |  |  |  |  |
| No | Reference | Reference | Reference | Reference |
| Yes | 01.02 (0.66, 1.58) | 1.03 (0.54, 1.99) | 1.15 (0.65, 2.05) | 1.48 (0.85, 2.57) |
| Body mass index at enrolments |  |  |  |  |
| Per 1-unit increase | 1.02 (0.95, 1.10) | 1.06 (0.95, 1.17) | 1.00 (0.92, 1.08) | 1.01 (0.95, 1.08) |
| **Child characteristics** |  |  |  |  |
| Sex |  |  |  |  |
| Male | Reference | Reference | Reference | Reference |
| Female | 0.99 (0.66, 1.46) | 0.90 (0.50, 1.64) | 0.98 (0.64, 1.49) | 1.07 (0.70, 1.63) |
| Gestational age at birth |  |  |  |  |
| Per 1-unit increase | 1.00 (0.87, 1.13) | 1.00 (0.79, 1.26) | 1.05 (0.90, 1.22) | 0.90 (0.78, 1.04) |
| Birth weight |  |  |  |  |
| Per 500-unit increase | **0.77 (0.61, 0.97)*** | 1.36 (0.96, 1.91) | 0.99 (0.77, 1.27) | 1.11 (0.87, 1.43) |
| Ethnic origin |  |  |  |  |
| Western | Reference | Reference | Reference | Reference |
| Turkish and Moroccan | 1.15 (0.58, 2.28) | 0.56 (0.15 2.07) | 0.98 (0.46, 2.09) | 0.99 (0.47, 2.10) |
| African | 0.96 (0.55, 1.69) | 1.41 (0.61, 3.26) | 1.37 (0.75, 2.48) | 1.01 (0.56, 1.82) |
| Asian | 0.72 (0.37, 1.41) | 0.52 (0.12, 2.13) | 1.06 (0.53, 2.10) | 1.43 (0.74, 2.78) |
| Day care attendance until age 1 year |  |  |  |  |
| No | Reference | Reference | Reference | Reference |
| Yes | 1.19 (0.66, 2.17) | 0.82 (0.39, 1.73) | 0.85 (0.46, 1.56) | 0.95 (0.55, 1.65) |
| Asthma ever at age 10 years |  |  |  |  |
| No | Reference | Reference | Reference | Reference |
| Yes | 1.00 (0.56, 1.80) | 1.09 (0.49, 2.41) | 0.51 (0.25, 1.01) | 1.11 (0.61, 2.05) |
| Eczema ever at age 10 years |  |  |  |  |
| No | Reference | Reference | Reference | Reference |
| Yes | 1.57 (0.97, 2.54) | **2.93 (1.49, 5.75)**** | **2.22 (1.36, 3.62)**** | **1.77 (1.09, 2.88)*** |

Values are odds ratios (95% confidence interval) from logistic regression models based on imputed data. Models are adjusted for all characteristics. **^1^**Additionally adjusted for inhalant allergic sensitization. *P-value <0.05. **P-value <0.003.
