# Supplementary material for: Parental and child factors associated with inhalant and food allergy in a population-based prospective cohort study: the Generation R Study
Source: Eur J Pediatr. 2019 Aug 15;178(10):1507–17. doi: 10.1007/s00431-019-03441-5 (PMC6733817; doi:10.1007/s00431-019-03441-5)
Supplement: Supplementary file 6 — (DOCX 17 kb) [file 431_2019_3441_MOESM6_ESM.docx]

**Supplementary Table 5.** Associations of maternal, paternal and child characteristics with specific physician-diagnosed inhalant allergies in children at age 10 years.

|  | **Odds ratio (95% CI) for specific physician-diagnosed inhalant allergy** | | | |
| --- | --- | --- | --- | --- |
|  | **House dust mite^1^**  **n = 4,733** | **Hay fever^1^**  **n = 4,743** | **Cat^1^**  **n = 4,711** | **Dog^1^**  **n = 4,696** |
| **Maternal characteristics** |  |  |  |  |
| Age at enrolment |  |  |  |  |
| Per 1-unit increase | 0.96 (0.93, 1.00) | 0.99 (0.95, 1.03) | 0.99 (0.94, 1.03) | 0.98 (0.92, 1.03) |
| History of allergy, eczema or asthma |  |  |  |  |
| No | Reference | Reference | Reference | Reference |
| Yes | **1.46 (1.11, 1.91)** | **1.49 (1.14, 1.94)*** | 1.27 (0.92, 1.76) | 1.25 (0.81, 1.91) |
| Parity |  |  |  |  |
| 0 | Reference | Reference | Reference | Reference |
| ≥1 | 1.09 (0.82, 1.45) | **0.66 (0.50, 0.88)*** | 0.88 (0.62, 1.26) | 1.02 (0.65, 1.59) |
| Pet keeping during pregnancy |  |  |  |  |
| No | Reference | Reference | Reference | Reference |
| Yes | 0.94 (0.70, 1.24) | **0.66 (0.48, 0.91)*** | 0.86 (0.58, 1.25) | 0.81 (0.49, 1.35) |
| Body mass index at enrolment |  |  |  |  |
| Per 1-unit increase | 1.01 (0.97, 1.04) | 1.00 (0.96, 1.03) | 0.97 (0.93, 1.01) | 0.98 (0.93, 1.03) |
| **Paternal characteristics** |  |  |  |  |
| Age at enrolment |  |  |  |  |
| Per 1-unit increase | 1.02 (0.99, 1.05) | 1.00 (0.96, 1.03) | 1.01 (0.97, 1.05) | 1.01 (0.97, 1.07) |
| History of allergy, eczema or asthma |  |  |  |  |
| No | Reference | Reference | Reference | Reference |
| Yes | **1.73 (1.29, 2.30)**** | **1.72 (1.24, 2.39)**** | **2.07 (1.43, 2.99)**** | **1.95 (1.22, 3.11)*** |
| Body mass index at enrolment |  |  |  |  |
| Per 1-unit increase | 0.99 (0.93, 1.04) | 1.04 (0.98, 1.09) | 1.01 (0.95, 1.07) | 1.01 (0.94, 1.08) |
| **Child characteristics** |  |  |  |  |
| Sex |  |  |  |  |
| Male | Reference | Reference | Reference | Reference |
| Female | 0.84 (0.65, 1.09) | **0.58 (0.45, 0.75)**** | 0.77 (0.56, 1.06) | **0.57 (0.38, 0.87)** |
| Gestational age at birth |  |  |  |  |
| Per 1-unit increase | 1.04 (0.95, 1.14) | **0.90 (0.83, 0.98)*** | 1.03 (0.92, 1.16) | 1.15 (0.99, 1.33) |
| Birth weight |  |  |  |  |
| Per 500-unit increase | 1.03 (0.88, 1.19) | 1.08 (0.93, 1.25) | 0.98 (0.81, 1.18) | 0.86 (0.68, 2.86) |
| Ethnic origin |  |  |  |  |
| Western | Reference | Reference | Reference | Reference |
| Turkish and Moroccan | **1.65 (1.05, 2.61)*** | 1.48 (0.91, 2.40) | 1.56 (0.88, 2.77) | 1.40 (0.68, 2.86) |
| African | 1.20 (0.78, 1.86) | 1.21 (0.78, 1.87) | 0.87 (0.50, 1.52) | 1.17 (0.63, 2.19) |
| Asian | 1.23 (0.75, 2.01) | 1.23 (0.75, 2.01) | **0.18 (0.06, 0.50)**** | **0.32 (0.11, 0.89)*** |
| Day care attendance until age 1 year |  |  |  |  |
| No | Reference | Reference | Reference | Reference |
| Yes | 1.09 (0.71, 1.66) | 1.03 (0.72, 1.48) | 1.04 (0.69, 1.55) | 0.90 (0.50, 1.61) |
| Asthma ever at age 10 years |  |  |  |  |
| No | Reference | Reference | Reference | Reference |
| Yes | **6.06 (4.53, 8.10)**** | **2.86 (2.09, 3.92)**** | **5.28 (3.67, 7.59)**** | **5.95 (3.83, 9.24)**** |
| Eczema ever at age 10 years |  |  |  |  |
| No | Reference | Reference | Reference | Reference |
| Yes | **2.16 (1.65, 2.82)**** | **2.68 (2.03, 3.54)**** | **2.49 (1.77, 3.50)**** | **2.21 (1.43, 3.41)**** |

Values are odds ratios (95% confidence interval) from logistic regression models based on imputed data. Models are adjusted for all characteristics. **^1^**Additionally adjusted for physician-diagnosed food allergy. *P-value <0.05. **P-value <0.003.
