# Supplementary material for: Parental and child factors associated with inhalant and food allergy in a population-based prospective cohort study: the Generation R Study
Source: Eur J Pediatr. 2019 Aug 15;178(10):1507–17. doi: 10.1007/s00431-019-03441-5 (PMC6733817; doi:10.1007/s00431-019-03441-5)
Supplement: Supplementary file 7 — (DOCX 16 kb) [file 431_2019_3441_MOESM7_ESM.docx]

**Supplementary Table 6.** Associations of maternal, paternal and child characteristics with specific physician-diagnosed food allergies in children at age 10 years.

|  | **Odds ratio (95% confidence interval) for specific physician-diagnosed food allergy** | |
| --- | --- | --- |
|  | **Cashew nut^1^**  **n = 4,625** | **Peanut^1^**  **n = 4,678** |
| **Maternal characteristics** |  |  |
| Age at enrolment |  |  |
| Per 1-unit increase | 1.07 (0.98, 1.18) | 1.04 (0.97, 1.12) |
| History of allergy, eczema or asthma |  |  |
| No | Reference | Reference |
| Yes | 1.31 (0.65, 2.65) | 0.95 (0.55, 1.65) |
| Parity |  |  |
| 0 | Reference | Reference |
| ≥1 | 1.06 (0.54, 2.08) | 0.94 (0.54, 1.66) |
| Pet keeping during pregnancy |  |  |
| No | Reference | Reference |
| Yes | 0.82 (0.36, 1.87) | 1.07 (0.60, 1.91) |
| Body mass index at enrolment |  |  |
| Per 1-unit increase | 0.97 (0.85, 1.11) | 0.98 (0.91, 1.05) |
| **Paternal characteristics** |  |  |
| Age at enrolment |  |  |
| Per 1-unit increase | 0.97 (0.89, 1.07) | 0.99 (0.92, 1.06) |
| History of allergy, eczema or asthma |  |  |
| No | Reference | Reference |
| Yes | 1.03 (0.49, 2.14) | 1.56 (0.86, 2.81) |
| Body mass index at enrolment |  |  |
| Per 1-unit increase | 0.97 (0.85, 1.11) | 0.96 (0.87, 1.06) |
| **Child characteristics** |  |  |
| Sex |  |  |
| Male | Reference | Reference |
| Female | 1.00 (0.53, 1.89) | 1.08 (0.64, 1.82) |
| Gestational age at birth |  |  |
| Per 1-unit increase | 0.92 (0.74, 1.15) | 0.98 (0.82, 1.17) |
| Birth weight |  |  |
| Per 500-unit increase | 0.94 (0.66, 1.35) | 0.95 (0.71, 1.28) |
| Ethnic origin |  |  |
| Western | Reference | Reference |
| Turkish and Moroccan | 0.62 (0.17, 2.29) | 0.58 (0.17, 1.97) |
| African | 2.01 (0.83, 4.86) | 1.63 (0.76, 3.50) |
| Asian | 2.13 (0.74, 6.11) | **2.42 (1.08, 5.42)*** |
| Day care attendance until age 1 year |  |  |
| No | Reference | Reference |
| Yes | 0.45 (0.18, 1.16) | 0.86 (0.42, 1.75) |
| Asthma ever at age 10 years |  |  |
| No | Reference | Reference |
| Yes | 1.44 (0.63, 3.31) | 1.85 (0.94, 3.63) |
| Eczema ever at age 10 years |  |  |
| No | Reference | Reference |
| Yes | **7.36 (3.20, 16.94)** | **5.58 (3.08, 10.10)**** |

Values are odds ratios (95% confidence interval) from logistic regression models based on imputed data. Models are adjusted for all characteristics. **^1^**Additionally adjusted for physician-diagnosed inhalant allergy. *P-value <0.05. **P-value <0.003.
